# Hemoglobin Concentration May Affect the Effect of Atorvastin on Chronic Subdural Hematoma After Burr-Hole Drainage at High Altitude

**DOI:** 10.3389/fnins.2020.00503

**Published:** 2020-06-12

**Authors:** Linjie Wei, Chi Lin, Mingfeng Zhong, Jianbo Zhang, Gang Zhu

**Affiliations:** ^1^Department of Neurosurgery, Southwest Hospital, Third Military Medical University (Army Medical University), Chongqing, China; ^2^Department of Neurosurgery, PLA 956th Hospital, Linzhi, China; ^3^Department of Neurosurgery, First People’s Hospital of Honghe City, Yunnan, China; ^4^Department of Function, The People’s Hospital of Weiyuan County, Sichuan, China; ^5^Department of Neurosurgery, The General Hospital of Southern Theater Command PLA, Guangzhou, China

**Keywords:** high altitude, atorvastatin, chronic subdural hematoma, burr hole, hemoglobin concentration

## Abstract

**Objective:**

Chronic subdural hematoma (CSDH) is a common disease. Atorvastatin calcium can increase CSDH absorption. However, whether atorvastatin can increase hematoma absorption and reduce recurrence at high altitudes is not clear.

**Methods:**

After burr-hole drainage, CSDH patients were divided into an atorvastatin group and a control group. Follow-up computed tomography (CT) was performed on day 1, months 1, 2, and 3 after surgery. Then, the recurrence rate, poor therapeutic effect, time to recurrence, poor surgical result, recurrence with operation, CSDH volume, and Markwalder grading scale score (MGSS) were calculated, and related risk factors were analyzed.

**Results:**

The non-recurrent and recurrent patients in the control group differed significantly in terms of the hemoglobin concentration (HB) [176.24 ± 16.43 vs. 194.25 ± 12.34 (g/L), *p* < 0.01], CT value [41.92 ± 10.76 vs. 34.12 ± 8.78 (Hu), *p* < 0.01], and low-density time [3.88 ± 1.04 vs. 5.50 ± 0.87 (d), *p* < 0.01]. The non-recurrent and recurrent patients in the atorvastatin group differed significantly in terms of the HB [172.66 ± 16.41 vs. 190.45 ± 10.23 (g/L), *p* < 0.01], CT value [38.91 ± 7.16 vs. 29.50 ± 8.61 (Hu), *p* < 0.01], and mixed [2 vs. 4 (n), *p* < 0.05] and low-density time [4.09 ± 0.75 vs. 5.45 ± 1.12 (d), *p* < 0.01]. The logistic regression analysis showed that HB [odds ratio, 1.14; 95% confidence interval (CI), 1.04–1.25 in the control group, odds ratio, 1.13; 95% CI, 1.03–1.23 in the atorvastatin group] and low-density time (odds ratio, 3.53; 95% CI, 1.42–8.74 in the control group, odds ratio, 2.53; 95% CI, 1.10–5.80 in the atorvastatin group) were possible risk factors for the two groups. The receiver operating characteristic curves showed that the area under the receiver operating characteristic curve values for the HB, CT value (Hu), and low-density time were 0.812, 0.702, and 0.755 for all subjects; 0.812, 0.719, and 0.790 for the control group; and 0.807, 0.682, and 0.756 for the atorvastatin group, respectively. The postoperative follow-up results showed that there was no significant difference in the recurrence rate, poor therapeutic effect, time to recurrence, poor surgical result, recurrence with operation, CSDH volume, or MGSS between the two groups.

**Conclusion:**

The effect of atorvastatin was not significant after the operation. The risk factors for CSDH recurrence were the HB and low-density time. The HB was the most specific and sensitive predictor of CSDH recurrence.

## Introduction

Chronic subdural hematoma (CSDH) is a common and frequently occurring disease, and the incidence rises with age (Sahyouni et al., 2017). Burr-hole drainage is a routine operation for the treatment of CSDH (Tang et al., 2018; Soleman et al., 2019; Khan et al., 2019), but the recurrence rate is ∼10–39% (Kim et al., 2014; Jang et al., 2015). Local inflammatory responses may contribute to the recurrence of CSDH (Jaiswal and Sontakke, 2012; Zhang et al., 2020). Atorvastatin is an inhibitor of HMG-CoA reductase, and it can inhibit the biosynthesis of hydroxymethyl glutaryl coenzyme a reductase and cholesterol. In recent years, studies have shown that CSDH absorption significantly increases and that the recurrence rate is reduced with the use of atorvastatin (Tang et al., 2018; Quan et al., 2019; He et al., 2020). Many studies have investigated the effect of atorvastatin on CSDH in low-altitude regions, but few studies have been carried out in high-altitude regions. However, whether atorvastatin can increase hematoma absorption and reduce recurrence is unclear at high altitudes. In addition, high-altitude areas can lead to increased HB and coagulation dysfunction due to hypoxia (Xie et al., 2015). It is not clear whether this will affect CSDH absorption with atorvastatin calcium. Thus, the effect of atorvastatin treatment on CSDH was retrospectively summarized after burr-hole drainage at high altitudes.

## Materials and Methods

### Patient Selection

**FIGURE 1:**
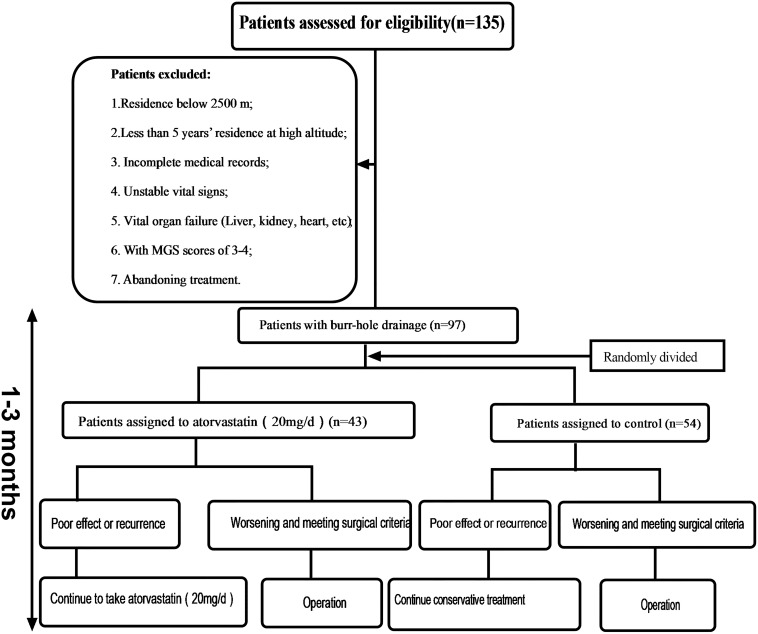
A flowchart for the identification process of eligible patients.

This study was approved by the ethics committee of the Southwest Hospital. Patient consent was waived by our review board. Case data are retrospective studies. From February 2013 to December 2018, a total of 135 patients were enrolled who had CSDH as determined by computed tomography (CT) (GE Lightspeed 64, Tokyo, Japan) scans and related examinations. Thirty-eight patients were excluded according to the following criteria (Figure 1): surgical indication: (1) the displacement of the midline was more than 0.8 cm; (2) the ipsilateral ventricle was obviously compressed or had disappeared; (3) the hematoma volume was more than 50 mL; (4) if the head CT did not reveal one of the first three criteria, the Markwalder grading scale score (MGSS) was 1–2. A total of 97 patients were treated with burr-hole drainage. Those patients were divided into the atorvastatin group and the control group, depending on whether atorvastatin was administered after the operation. The relevant data of the two groups were collected and recorded in detail upon admission, including sex, age, trauma history, medication history, hemoglobin concentration (HB), hematoma location, hematoma density, hematoma size, neurological function, symptom improvement, and comorbidity. Considering the risk of severe CSDH with increased intracranial pressure and prognosis, according to the literature (Wang et al., 2014), grades 0–3 CSDH was included, but this study enrolled only patients with grades 0–2 CSDH at high altitude. Figure 1 shows the steps for patient review. All data were independently and blindly reviewed by two senior neurosurgeons.

### Operation Method

**FIGURE 2:**
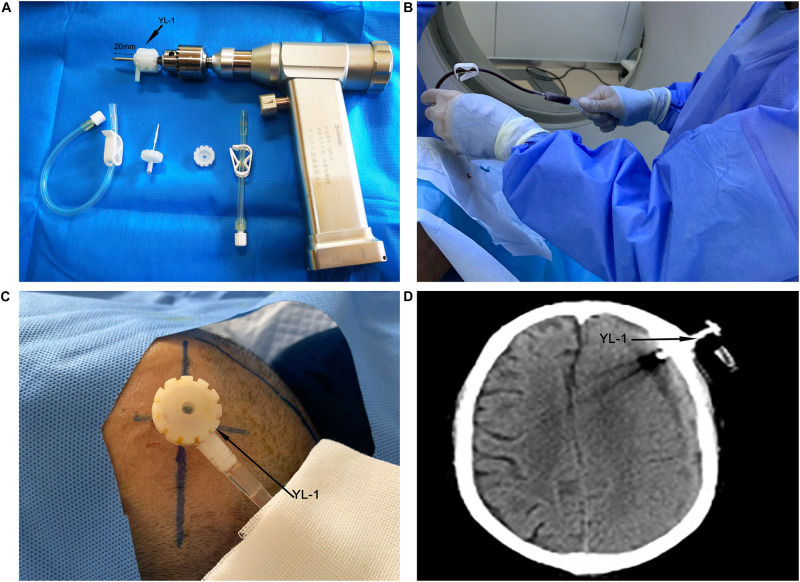
Yl-1 drainage tube (black arrow) before surgery, drill length 20 mm (two black arrows) **(A)**. YL-1 drainage tube was used to draw and rinse the CSDH during the operation **(B)**. YL-1 drainage tube (black arrow) continued to drain the hematoma after the operation **(C)**. YL-1 drainage tube (black arrow) on postoperative CT **(D)**.

**FIGURE 3:**
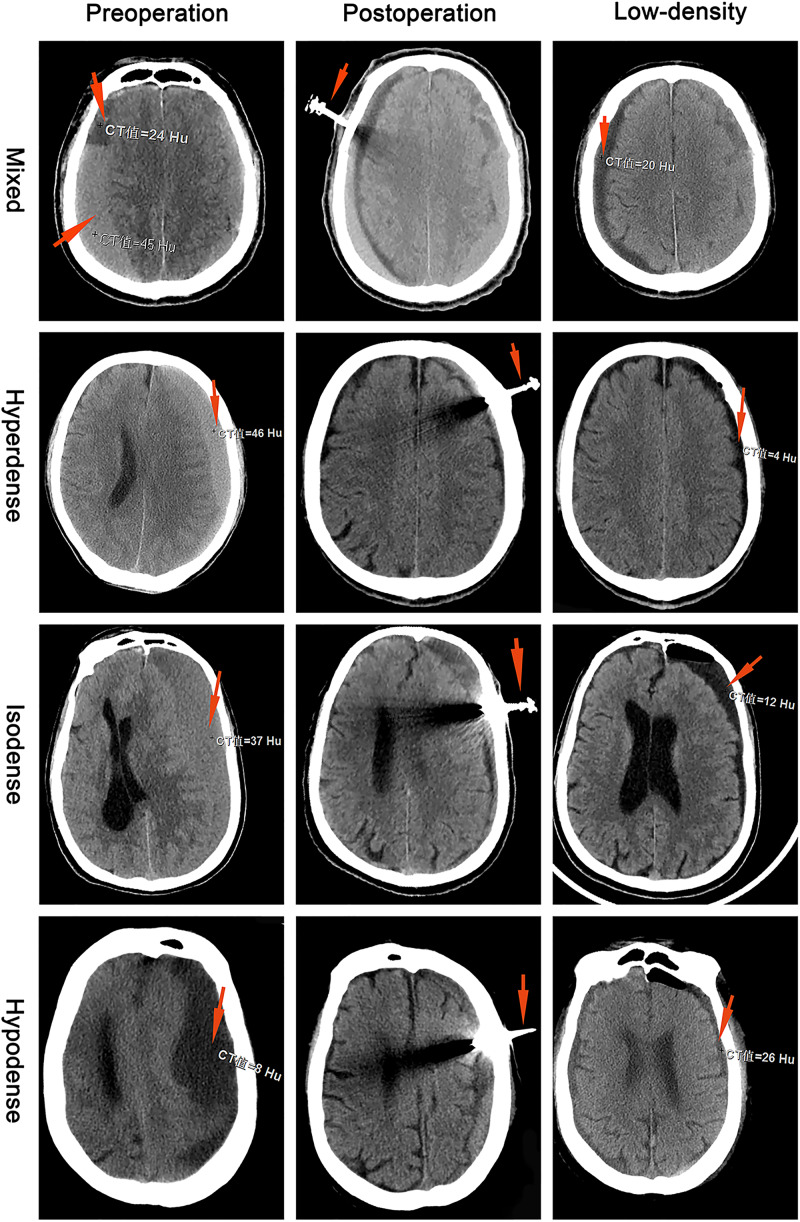
Chronic subdural hematoma classification: mixed, hyperdense, isodense, and hypodense. The preoperative red arrows show the CT values (Hu) for different CSDH types. The postoperative red arrow shows the YL-1 drainage tube. The low-density red arrow shows that the CT value (Hu) of the CSDH was less than 30.

According to the CT of the head, the largest hematoma plane was selected as the positioning point for drilling. All patients were placed under local anesthesia, and a 20 mm YL-1 puncture needle (Beijing Wan Tie Fu Medical Apparatus Co. Ltd., Beijing, China) was used for drilling after selecting the positioning point. The positioning point was strictly and slowly rinsed with warm normal saline until the rinsing fluid was clear (Figure 2). The follow-up head CT examination found obvious brain tissue expansion, subdural hematoma ≤50 mL in volume, and low-density CSDH [CT value (Hu) ≤ 30: equal to CT value (Hu) of hypodense hematoma] on days 2–6 after the operation, and the drainage tube was removed (Figure 3). After 6 days, subdural hematoma was not < 50 mL in volume, and there was no low-density CSDH [CT value (Hu) ≤ 30]. The drainage tube was also pulled out, and the results were recorded as poor.

### Design and Atorvastatin Therapy

There were 43 patients in the atorvastatin group and 54 in the control group. Patients were randomly assigned to two groups. The patients took a once-daily oral dose of 20 mg atorvastatin for 3 months (Lipitor; Pfizer, Inc., Dalian, China) in the atorvastatin group. If the CSDH disappeared within 3 months, atorvastatin was stopped. The patients were not given atorvastatin in the control group; in addition to atorvastatin, other treatments and drugs were the same in both groups. All patients were followed up regularly for 3 months.

### Evaluation and Follow-Up

**FIGURE 4:**
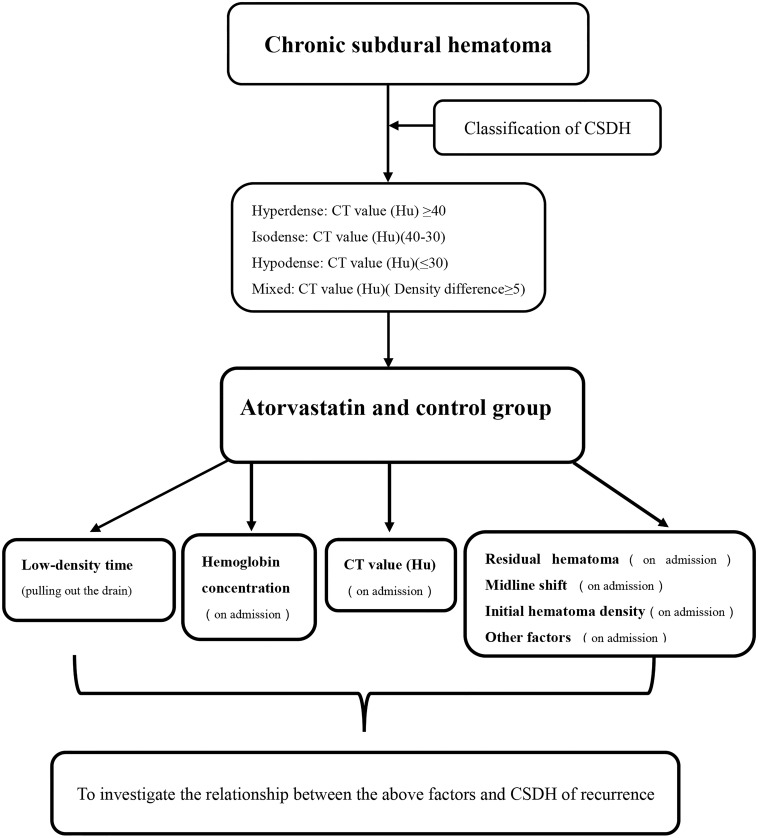
Steps to test the indicators for CSDH.

Each patient had a head CT scan to measure hematoma volume before and after the treatment. Hematoma volume was calculated based on ImageJ software (Bethesda, Maryland, USA) as previously described (Hanell et al., 2012). Chronic subdural hematoma was classified as mixed, hypodense, isodense, or hyperdense, according to the CT value (Hu) (Figure 4). If it was a mixed hematoma, the high-, medium-, and low-density hematoma CT values (Hu) were calculated and then averaged. The MGSS and Glasgow Coma Scale grades were obtained before the treatment (Table 1). The time for the CSDH to become low density was recorded after the operation (Figure 3). Follow-up CT was performed on the first day, months 1, 2, and 3 after the operation, and then the time to recurrence, recurrence with operation, poor therapeutic effect, poor surgical result, CSDH volume, and MGSS were calculated. If the patient’s condition worsened, additional CT scans were performed. The definition of non-recurrence was CSDH volume ≤ 50 mL, CT value (Hu) ≤ 30, and MGSS < 1: (1) poor effect: although the hematoma did not reach the upper recurrence standard, the absorption was poor [hematoma volume (third month) divided by hematoma volume (upon admission) ≥ 50%]; (2) good effect: complete disappearance of hematoma or hematoma volume (third month) < 50%. The definition of recurrence was CSDH volume > 50 mL, CT value (Hu) > 30, or symptoms of neurological dysfunction that appeared again (MGSS ≥ 1) (Abouzari et al., 2007). If recurrent CSDH was consistent with surgical indications, surgical treatment was performed with a YL-1 puncture needle. Logistic regression analysis of risk factors and receiver operating characteristic (ROC) curves were performed for non-recurrent and recurrent patients using the relevant data upon admission. If the patient’s recovery was poor or if there was hematoma recurrence, treatment was carried out as shown in Figure 1, but regardless of the outcome of the two groups of patients, the treatment effect was evaluated at the third month.

**TABLE 1:** Simplified GCS and MGSS for the neurological conditions of the patients with CSDH.

**GCS**	**MGSS**	**Symptoms**
15	0	No neurological symptoms
14–12	1	Mild symptoms such as dizziness and headache, mild neurological disorders such as asymmetric tendon reflexes
11–9	2	Lethargy, misorientation, severe neurological impairment such as mild hemiplegia
8–6	3	Shallow coma with appropriate response to pain stimuli, severe neurological dysfunction such as hemiplegia
5–3	4	Coma with a lack of response to pain stimuli, decerebrate, or decorticate posturing

### Statistical Analysis

All analyses were performed using SPSS 19 (IBM Corp., Armonk, NY, United States). Normally distributed data are expressed as the mean ± standard deviation. Quantitative data were analyzed between the two groups using separate *t*-tests and analysis of variance, whereas categorical data were compared using χ^2^-tests between the atorvastatin and control groups. The risk factors for the atorvastatin and control groups were analyzed by a single factor. Logistic regression was used to analyze risk factors between the atorvastatin group and the control group. Receiver operating characteristic curve analysis was used to investigate the diagnostic relationship between risk factors and hematoma recurrence. The Youden index was used to determine the optimal cutoff value as previously described. Differences were considered significant at *P* < 0.05. All data analyses were conducted by two investigators in a blinded manner.

## Results

### Participant Characteristics

A total of 135 patients with CSDH were enrolled in the study. Thirty-eight patients were excluded from the study (28.15%) (Figure 1). Among them, 43 patients received oral atorvastatin after the operation. There were no significant differences in the clinical or radiological characteristics between the atorvastatin group and the control group.

### Risk Factors for CSDH Recurrence

**FIGURE 5:**
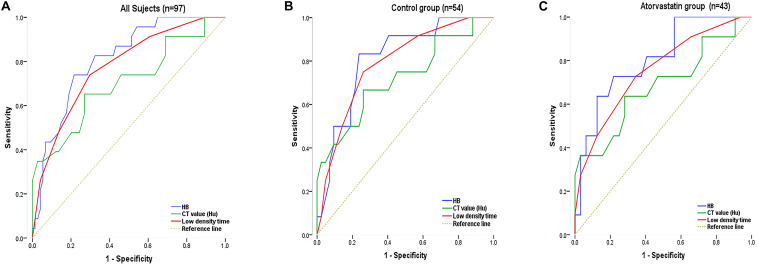
The ROC curve was used to measure the HB, CT value (Hu), and low-density time for predicting the cutoff value of recurrence after surgery. Specificity and sensitivity were used as measures of CSDH recurrence accuracy. Analyses were performed for all subjects in the sample (**A**; *n* = 97) and separately for the control (**B**; *n* = 54) and atorvastatin (**C**; *n* = 43) groups. Chronic subdural hematoma classification recurrence was estimated using the AUC **(A–C)**.

The non-recurrent and recurrent patients in the control group differed significantly in terms of the HB [176.24 ± 16.43 vs. 194.25 ± 12.34 (g/L), *p* < 0.01], CT value [41.92 ± 10.76 vs. 34.12 ± 8.78 (Hu), *p* < 0.01], and low-density time [3.88 ± 1.04 vs. 5.50 ± 0.87 (d), *p* < 0.01] (Table 2). The non-recurrent and recurrent patients in the atorvastatin group differed significantly in terms of the HB [172.66 ± 16.41 vs. 190.45 ± 10.23 (g/L), *p* < 0.01], CT value [38.91 ± 7.16 vs. 29.50 ± 8.61 (Hu), *p* < 0.01], and mixed [2 vs. 4 (n), *p* < 0.05] and low-density time [4.09 ± 0.75 vs. 5.45 ± 1.12 (d), *p* < 0.01] (Table 2). The logistic regression analysis showed the comparison of the HB [odds ratio, 1.138; 95% confidence interval (CI), 1.038–1.247], CT value (Hu) (odds ratio, 1.127; 95% CI, 0.995–1.277), and low-density time (odds ratio, 3.527; 95% CI, 1.420–8.735) in the control group (Table 3). The HB (odds ratio, 1.126; 95% CI, 1.029–1.233), CT value (Hu) (odds ratio, 1.062; 95% CI, 0.975–1.156), and low-density time (odds ratio, 2.259; 95% CI, 1.103–5.799) were compared in the atorvastatin group (Table 3). The HB and low-density time might be risk factors for the recurrence of CSDH. The ROC curves were used to measure the HB, CT value (Hu), and low-density time for predicting the cutoff value of recurrence after the operation. Specificity and sensitivity were also used as measures of CSDH recurrence accuracy. Receiver operating characteristic curve analysis showed that the area under the ROC curve (AUC) values for the HB, CT value (Hu), and low-density time were 0.812, 0.702, and 0.755 for all subjects (Table 4 and Figure 5A); 0.812, 0.719, and 0.790 for the control group (Table 4 and Figure 5B); and 0.807, 0.682, and 0.756 for the atorvastatin group (Table 4 and Figure 5C), respectively. The specificity and sensitivity for HB were higher than those for the CT value (Hu) and low-density time (Table 4 and Figures 5A–C).

**TABLE 2:** Characteristics of patients with CSDH included in the control and atorvastatin groups.

	**Control group**			**Atorvastatin group**		
	**No recurrence (*n* = 42)**	**Recurrence (*n* = 12)**	***p* value**	**No recurrence (*n* = 32)**	**Recurrence (*n* = 11)**	***p* value**
Age (y)	60.00 ± 13.66	61.14 ± 16.36	*p* > 0.05	64.25 ± 12.71	62.82 ± 13.51	*p* > 0.05
Female sex	7	2	*p* > 0.05	7	3	*p* > 0.05
Altitude	2939.00 ± 388.29	2986.82 ± 366.74	*p* > 0.05	3085.69 ± 343.66	3049.33 ± 389.29	*p* > 0.05
Smoking	10	3	*p* > 0.05	7	4	*p* > 0.05
Alcohol use	13	3	*p* > 0.05	10	5	*p* > *0.05*
**Comorbidities**						
Diabetes (n)*	5	1	*p* > 0.05	3	1	*p* > 0.05
Hypertensio n (n)	8	3	*p* > 0.05	8	4	*p* > 0.05
Heart disease (n)*	5	0	*p* > 0.05	2	0	*p* > *0.05*
Pulmonary disease (n)*	1	1	*p* > 0.05	2	1	*p* > 0.05
Other diseases (n)	7	2	*p* > 0.05	6	1	*p* > 0.05
**Drug history**						
Antiplatelet treatment, No	6	1	*p* > 0.05	5	1	*p* > 0.05
Antiplatelet treatment, No*	3	0	*p* > 0.05	3	0	*p* > 0.05
**MGSS (Upon admission)**						
M_0_	0	0	*p* > 0.05	0	0	*p* > *0.05*
M_1_	20	5	*p* > 0.05	18	3	*p* > 0.05
M_2_	22	7	*p* > 0.05	15	7	*p* > 0.05
**GCS (Upon admission)**						
9 (n)*	1	0	*p* > 0.05	0	1	*p* > *0.05*
10–12 (n)*	2	1	*p* > 0.05	4	1	*p* > 0.05
13–15 (n)	39	11	*p* > 0.05	28	9	*p* > 0.05
Platelet (×10^9^)	212.79 ± 54.06	216.92 ± 82.02	*p* > 0.05	232.46 ± 69.06	209.34 ± 55.51	*p* > 0.05
Blood glucose (mmol/L)	6.03 ± 2.59	6.30 ± 0.93	*p* > 0.05	6.22 ± 1.74	5.96 ± 1.19	*p* > 0.05
HB (g/L)	176.24 ± 16.43	194.25 ± 12.34	*p* < 0.01	172.66 ± 16.41	190.45 ± 10.23	*p* < 0.01
CSDH volume (ml)	87.82 ± 48.07	91.75 ± 40.15	*p* > 0.05	84.73 ± 56.32	91.57 ± 35.21	*p* > 0.05
CT value (Hu)	41.92 ± 10.76	34.12 ± 8.78	*p* < 0.01	38.91 ± 7.16	29.50 ± 8.61	*p* < 0.01
**Initial hematoma density**						
Hypodense (n)*	10	2	*p* > 0.05	7	3	*p* > 0.05
Isodense (n)*	17	3	*p* > 0.05	17	2	*p* > 0.05
Hyperdense (n)*	9	3	*p* > 0.05	6	2	*p* > 0.05
Mixed (n)*	6	4	*p* > 0.05	2	4	*p* < 0.05
**Initial hematoma laterality**						
(bilateral: unilateral)*	6:36	3:9	*p* > 0.05	6:26	2:9	*p* > 0.05
Midline shift ≥ 10 mm (n)	14	5	*p* > 0.05	12	4	*p* > 0.05
Low-density time (d)	3.88 ± 1.04	5.50 ± 0.87	*p* < 0.01	4.09 ± 0.75	5.45 ± 1.12	*p* < 0.01
Residual hematoma	10.73	10.27	*p* > 0.05	12.61	12.32	*p* > 0.05

*Fisher.

**TABLE 3:** Results of logistic regression analysis indicating predictors of risk factors.

**Group**	**Indicators**	**B**	**BE**	**Wald**	***P-*value**	**OR (95% CI)**
	HB	0.129	0.047	7.635	<0.01	1.138 (1.038–1.247)
	CT Value (Hu)	0.119	0.064	3.511	>0.05	1.127 (0.995–1.277)
	Low-density time	1.260	0.463	7.422	<0.01	3.527 (1.42–8.735)
	HB	0.119	0.046	6.601	<0.01	1.126 (1.029–1.233)
	CT Value (Hu)	0.60	0.44	1.904	>0.05	1.062 (0.975–1.156)
	Low-density time	0.928	0.423	4.800	<0.05	2.529 (1.103–5.799)

**TABLE 4:** Indices of lowest relative factors for predicting recurrence.

	**All subjects (*n* = 97)**			**Control group (*n* = 54)**			**Atorvastatin group (*n* = 43)**		
			
	**HB**	**CT value (Hu)**	**Low-density time**	**HB**	**CT value (Hu)**	**Low-density time**	**HB**	**CT value (Hu)**	**Low-density time**
Cut-off value (%)	52.3%	38.2%	44.2%	59.5%	40.5%	48.9%	51.1%	35.5%	38.4%
AUC	0.812	0.702	0.755	0.812	0.719	0.79	0.807	0.682	0.756
95% CI	(0.720–0.905)	(0.569–0.835)	(0.668–0.882)	(0.684–0.941)	(0.540–0.898)	(0.650–0.929)	(0.662–0.952)	(0.481–0.883)	(0.568–0.925)
Sensitivity (%)	73.9%	65.2%	73.9%	83.3%	66.7%	75%	72.7%	63.6%	72.7%
Specificity (%)	78.4%	73%	72.1%	76.2%	73.8%	73.8%	62.5%	71.9%	65.6%
*P*-value	<0.01	>0.05	<0.05	<0.01	<0.05	<0.01	<0.01	>0.05	<0.05

### Postoperative Follow-Up Results

Table 5 summarizes the outcomes in the control and atorvastatin groups. The recurrence rate (22.22%) found in the control group was significantly lower than the recurrence rate (25.58%) found in the atorvastatin group (*P* < 0.05) (Table 5). Chronic subdural hematoma recurrence was observed in 12 patients in the control group and 11 patients in the atorvastatin group (Table 5). The number of patients with recurrence after the operation was six in the control group and four in the atorvastatin group (Table 5). Poor effects were found in five patients in the control group and five patients in the atorvastatin group (Table 5). Poor surgical result was found in three patients in the control group and two patients in the atorvastatin group (Table 5). There was no significant difference in the other evaluations (Table 5). There was no difference in the CSDH volume or MGSS between the control and atorvastatin groups on day 1, months 1, 2, and 3 after the operation (*p* > 0.05) (Table 5).

**TABLE 5:** Follow-up effect analysis in the control and atorvastatin groups.

	**Control group *n* = 54**	**Atorvastatin group *n* = 43**	
**Non-recurrence**			
Poor effect	5	5	>0.05
Good effect	24	25	>0.05
Recurrence, No. (%)	12 (22.22%	11 (25.58%)	>0.05
Recurrence with operation*	6	4	>0.05
Poor surgical result*	3	2	>0.05
**Time to recurrence**			>0.05
1 month	9	10	>0.05
2 months*	2	1	>0.05
3 months*	1	0	>0.05
**CSDH volume (ML)**			
0 V	45.44 ± 5.65	43.12 ± 7.89	>0.05
1 V	25.72 ± 3.45	23.64 ± 5.42	>0.05
2 V	18.12 ± 2.53	19.41 ± 3.11	>0.05
3 V	15.22 ± 3.23	15.57 ± 2.32	>0.05
**MGSS**			
0M_0__–__1_	30	28	>0.05
0M_2_	23	15	>0.05
0M_3__–__4_*	1	0	>0.05
1M_0__–__1_	41	35	>0.05
1M_2_	13	9	>0.05
1M_3__–__4_	0	0	>0.05
2M_0__–__1_	46	38	>0.05
2M_2_	9	6	>0.05
2M_3__–__4_	0	0	>0.05
3M_0__–__1_	51	41	>0.05
3M_2_*	3	2	>0.05
3M_3__–__4_	0	0	>0.05

0, 1, 2, and 3 V represent the *CSDH* volume on the first day, months 1, 2, and 3 after surgery, respectively. 0, 1, 2, and 3 M represent the MGSS on the first day, months 1, 2, and 3 after surgery, respectively. Low-density time, Time for CSDH to achieve low density (CT value ≤ 30). *Fisher test.

## Discussion

This study was a retrospective study on the effect of atorvastatin on CSDH after burr-hole drainage at high altitude. The results showed that atorvastatin had no significant effect on hematoma absorption or on reducing the recurrence rate. It was found that the HB upon admission and the time for the hematoma to achieve a low density after the operation might have affected hematoma recurrence. However, because of the limited caseload, it may affect our conclusions. Burr-hole drainage is the first choice of treatment because of its limited amount of trauma, but the recurrence rate of hematoma is high. Studies have shown that atorvastatin can accelerate the absorption of postoperative CSDH and reduce the recurrence rate (Liu et al., 2016). Because of the special hypoxic environment, the HB increases at high altitudes. Hypoxia leads to brain inflammation (Zhou et al., 2017). Chronic subdural hematoma itself is a vascular response caused by chronic inflammation (Li et al., 2014). It has been speculated that the incidence of CSDH should be relatively high at high altitudes. In addition, brain injury is more serious, and neurological recovery is poor at high altitudes (Yu et al., 2014; Zhu et al., 2015; Wei et al., 2016, 2020). However, while we could not evaluate the prognosis of CSDH at high altitudes compared with low altitudes, our analysis showed that there was no difference in the CSDH volume or MGSS between the control group and the atorvastatin group on 1 day, 1, 2, and 3 months after the operation. What caused the poor therapeutic effect of atorvastatin calcium on CSDH at high altitude? The result might be because of the special hypoxic environment or high HB, which might affect the absorption of atorvastatin in hematoma. The findings might also be due to the fact that, with CSDH, the chronic inflammation of blood vessels is very serious, and the effect of atorvastatin calcium on the inhibition of the vascular response is poor. In addition, we retrospectively analyzed the risk factors for CSDH recurrence. The results showed that the risk factors for recurrence were HB and low-density time. We speculated that the higher the HB was, and the greater the hematoma inflammatory response, the more serious the vascular response around the hematoma cavity would be, and the greater the possibility of repeated bleeding. The longer the hematoma was absorbed below a CT value of 30, the higher the recurrence rate was, which might be due to the large inflammatory reaction of its own blood vessels and the possibility of repeated exudation. The longer the hematoma density was low [CT value (Hu) ≤ 30], the greater the chance of recurrence. At the same time, ROC curve analysis showed that the HB had high specificity and sensitivity. The ROC curve showed that the low-density time had a certain sensitivity for recurrence, but it was less sensitive than the HB. The ROC curve analysis was consistent with the logistic regression analysis: the HB and low-density time had statistical significance for hematoma recurrence. We analyzed the therapeutic effect of atorvastatin calcium on CSDH at high altitude. The results were not consistent with those at low altitude. However, we found some risk factors for recurrence from these limited case data, which might provide some methods for the future prevention of recurrence.

## Conclusion

The effect of atorvastatin was not obvious after the operation. The risk factors for CSDH recurrence were HB and low-density time. The HB was the most specific and sensitive factor for predicting recurrence upon admission.

### Limitations

This study was limited by the caseload and the retrospective design. These insufficiencies may limit the generalizability of our findings.

## Data Availability Statement

All datasets generated for this study are included in the article/supplementary material.

## Ethics Statement

The studies involving human participants were reviewed and approved by the ethics committee of the Southwest Hospital. The patients/participants provided their written informed consent to participate in this study.

## Author Contributions

GZ designed this study. CL and LW collected the case data. MZ and JZ revised the manuscript to its final draft. LW contributed important discussion and interpretation of the results. All authors read and approved the final version of this manuscript.

## Conflict of Interest

The authors declare that the research was conducted in the absence of any commercial or financial relationships that could be construed as a potential conflict of interest.

## Abbreviations

CT: computed tomography
CSDH: chronic subdural hematoma
SD: standard deviation
ROC: receiver operating characteristic
Hu: hounsfield unit
MGSS: Markwalder grading scale score
HB: hemoglobin concentration.
